# Classification of Chaotic Squeak and Rattle Vibrations by CNN Using Recurrence Pattern

**DOI:** 10.3390/s21238054

**Published:** 2021-12-02

**Authors:** Jaehyeon Nam, Jaeyoung Kang

**Affiliations:** 1 Future Automotive Intelligent Electronics Core Technology Center, Kongju National University, Cheonan 31080, Korea; jaehyeon86@kongju.ac.kr; 2 Department of Mechanical Engineering, Inha University, Incheon 22212, Korea

**Keywords:** squeak, rattle, convolutional neural network, Lyapunov exponent, chaos, recurrence patterns

## Abstract

The chaotic squeak and rattle (S&R) vibrations in mechanical systems were classified by deep learning. The rattle, single-mode, and multi-mode squeak models were constructed to generate chaotic S&R signals. The repetition of nonlinear signals generated by them was visualized using an unthresholded recurrence plot and learned using a convolutional neural network (CNN). The results showed that even if the signal of the S&R model is chaos, it could be classified. The accuracy of the classification was verified by calculating the Lyapunov exponent of the vibration signal. The numerical experiment confirmed that the CNN classification using nonlinear vibration images as the proposed procedure has more than 90% accuracy. The chaotic status and each model can be classified into six classes.

## 1. Introduction

Chaotic squeak and rattle (S&R) vibrations are a significant factor for evaluating the quality of automotive parts. Early S&R was detected with a find-and-fix approach by a subjective evaluation from engineers. Therefore, highly skilled experts are needed to detect S&R. In addition, the evaluation was made subjectively because of the differences in training and expertise and the use of different measurement tools. For this reason, an objective tool is needed for quantitative measurements. On the other hand, the S&R indices developed for such an evaluation must define the threshold value of the index, and the threshold value must be based on a subjective evaluation [1,2,3,4]. In addition, S&R problems occurring in mechanical systems are challenging to analyze because they include extreme nonlinearities, such as impact and friction [5,6,7,8,9].

Squeak is a self-excited vibration caused by friction that frequently occurs in automobile brakes, artificial hip joints, and gear systems [8,10,11]. Many studies have examined the vibration instability caused by friction based on an analysis of brake squill noise. The method to solve these problems was studied mainly by analyzing the instability using vibration equations, including nonlinearity of friction and linear stability through the linearization of nonlinear terms. On the other hand, the linearized approach can only be investigated near equilibrium. Kang [8] described complex models, such as instability caused by friction curves, modal coupling instability, gyroscopic, and friction damping occurring in automobile disc brake systems. They also analyzed the influence of squeal. Nam et al. [10] investigated the vibration instability in the lead screw system experimentally and analyzed the instability mechanism using the finite element method (FEM). Ouenzerfi et al. [11] examined the frictional instability occurring in an artificial hip joint and investigated the instability through a detailed FEM. In addition, the friction force is expressed as a function of the velocity in the dynamic instability of the friction-induced model. Higher-order nonlinear problems, such as chaos, were described because the model includes extreme nonlinearity in the creep section [12,13,14,15,16]. Kang [15] used a two-degree-of-freedom friction model to show that the chaotic phenomenon is generated by self-excited vibrations and investigated the parameters that create chaos.

Rattle can cause chaos due to extreme discontinuity caused by the impact force, including vibrations due to the impact vibrations induced by the excitation. For this reason, the dynamics of impact motion have been studied extensively for a study of chaos. Serweta et al. [17,18] examined the chaotic characteristics by calculating the Lyapunov exponent of an impact oscillator with symmetrical soft stops and rigid stop. Kang [19] analyzed the chaotic factors by calculating the Lyapunov exponent for the truncated number of modes of the impact beam under a distributed contact using the continuum beam model.

In addition to the theoretical approach, the analysis of such a nonlinear vibration signal has been performed using a visualization method and a quantified index. A general signal analysis method takes an FFT in the time series and analyzes the dynamic characteristics in the frequency domain. Furthermore, dynamical characteristics were examined through the trajectory of the attractor in the phase space. As many studies on signal analysis have been carried out, signal visualization methods, such as Gauss wavelets [20,21] and a recurrence plot (RP), have been developed. Marwan et al. [22] introduced various RP methods to visualize the dynamic characteristics in a complex system. RP is a power tool that visualizes and analyzes the recurrence characteristics of dynamic systems. In addition, recurrence can be visualized efficiently and developed formally using a matrix. The reciprocal of the longest diagonal of an RP is proportional to the largest Lyapunov exponent. This shows that RP can express both the recurrence and chaos characteristics well. An RP is represented on the reconstructed phase space that is determined using the time delay method [23,24,25,26]. Recurrence quantification analysis (RQA) can quantify the repetition characteristics through indices expressed as found in the recurrence rate (RR), the determinism (DET), and the average diagonal line length based on the RP. RQA is a good technique for quantifying recurrence properties, but the results are presented only in indices [22]. In addition, higher-order spectrum analysis (HOSA) and clustering techniques are used to analyze various methods, including high-dimensional nonlinearity [27].

Dynamics problems involving extreme nonlinearities, such as S&R, can be accompanied by chaos. The most accurate way to determine chaos is the Lyapunov exponent. Wolf et al. described a method called the spectrum of the largest Lyapunov exponent. A calculation algorithm was also developed [28], and chaos could be determined by parameter analysis. On the other hand, this algorithm cannot be applied in nonlinear dynamical systems, including discontinuities, and can only be used in smooth dynamical systems. In contrast, Muller’s algorithm can be applied to a non-smooth dynamical system through an indicator function and transition condition [16]. Determining the chaos through the Lyapunov exponents is advantageous if the governing equation for the system is known or the available observations are very long [29].

Recently, with the rapid development of artificial intelligence, many algorithms using machine learning have been developed. In particular, for image classification, numerous CNN models based on a convolutional neural network (CNN) have been established, and ResNet, which was released in 2015, transcends human cognitive ability [30]. Hsueh et al. [31] showed that the fault signal of a motor through the experiment could be classified in binary by a CNN. Nam et al. [32] reported that, even if the vibration signal includes discontinuous nonlinearities, such as impact, the chaotic signal can be classified by a CNN using the image visualized with an unthresholded RP. On the other hand, it only performed a dichotomous classification for chaos and non-chaos. Therefore, the binary classification study was extended to perform a multi-class classification of chaotic S&R vibration signals.

This study examined whether the rattle and squeak signals can be classified through a CNN, even if they are chaotic, by applying a signal visualization technique. Because CNN is an image-based classification technique, an RP-based dataset was constructed to express the repetition of a dynamic system quantitatively. A single-mode squeak, multi-mode squeak, and rattle model were built. A methodology that classifies six classes for the chaotic S&R model with high accuracy through a CNN is proposed.

## 2. Methods

**Figure 1:**
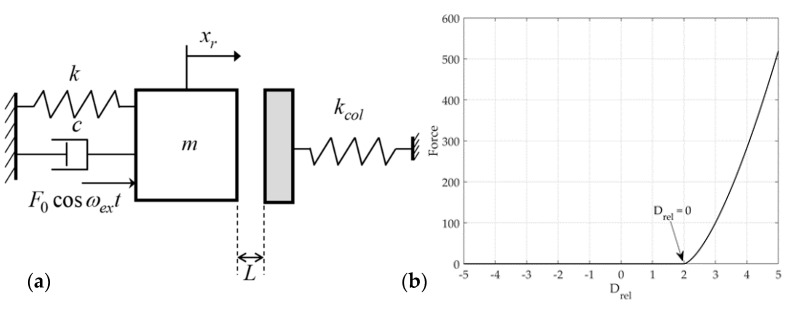
Rattle model: (**a**) 1-D model; (**b**) impact force.

In this study, a theoretical model of S&R vibration, a representative nonlinear vibration that can occur in a mechanical system, is used. Figure 1 presents the rattle model considering the mass, linear spring, nonlinear elastic contact, and damping. k is a linear spring coefficient. This system is excited with amplitude $f_{0}$ and excitation frequency $\omega_{ex}$. As shown in Figure 1a, the distance from the impact surface at the static equilibrium position of the system is L. In addition, the nonlinear elastic model of the impact force was defined as Hertz’s contact model [17,18]. The coordinate $x^{r}$ describes the vibration motion of the rattle model at the static equilibrium position. Figure 1b shows the impact force of Hertz’s contact model for a relative displacement. All systems consider a mass m attached to a spring with a stiffness coefficient k and coefficient of viscous damping c.

**Figure 2:**
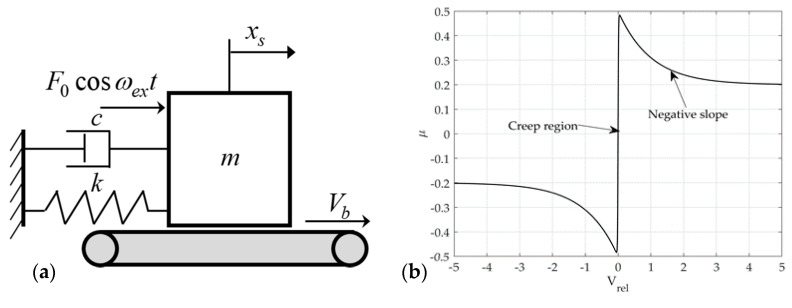
Single-mode squeak model: (**a**) 1-D model (**b**) friction–velocity curve.

**Figure 3:**
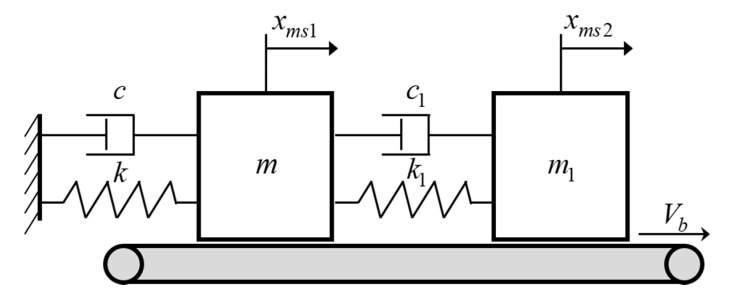
Multi-mode squeak model.

The single-mode squeak model is excited with the same amplitude $f_{0}$ and excitation frequency $\omega_{ex}$ as the rattle model shown in Figure 2a. On the contact surface, a frictional force is generated by the normal force and the relative velocity. The friction force includes the creep region and negative slope for the sliding speed from Coulomb’s law of friction, as shown in Figure 2b. The multi-mode squeak model has an added mass $m_{1}$, spring stiffness $k_{1}$, and coefficient of viscous damping $c_{1}$, and the frictional force generated by each mass is the same as that of the single-mode squeak model, as shown in Figure 3.

For the rattle model shown in Figure 1a

(1) $$m{\ddot{x}}_{r}+c{\dot{x}}_{r}+kx_{r}=F_{0}\cos\omega_{ex}t+F_{c}$$

where the contact model is the impact force by Hertz’s nonlinear elastic model as follows

(2) $$\begin{array}{ll} F_{c}=0 & \mathrm{if}\;x_{r}<L\\ F_{c}=k_{col}{\left(x_{r}-L\right)}^{3/2} & \mathrm{if}\;x_{r}\geq L \end{array}$$

Using the dimensionless time $\tau =t\sqrt{k/m}$ and the coordinate transformation $x_{r}(t)=X_{r}(\tau )$, the dimensionless equation of motion can be written as

(3) $${X^{\prime \prime }}_{r}+\beta {X^{\prime }}_{r}+X_{r}=f_{0}\cos\eta \tau +F_{c}\frac{1}{k}$$

where prime is differentiation for τ(≥0) and the dimensionless parameter is defined as $\Omega =\sqrt{k/m}$, $\eta =\omega_{ex}/\Omega$, $f_{0}=F_{0}/k$, $\beta =\sqrt{c^{2}/mk}$, $k_{c}=k_{col}\sqrt{f_{0}}/k$ and $u=X_{r}/f_{0}$. Therefore, the dimensionless equation of motion for the rattle model can be written as

(4) $$u^{\prime \prime }+\beta u^{\prime }+u=\cos\eta \tau +Hf_{c}$$

and the dimensionless form of the impact force is rewritten as follows

(5) $$\begin{array}{ll} f_{c}=0 & \mathrm{if}\;u<r\\ f_{c}=k_{c}{\left(u-r\right)}^{3/2} & \mathrm{if}\;u\geq r \end{array}$$

Equation of motion for the rattle model is expressed in vector form as follows

(6) $$u={\left[\begin{array}{cc} u & u^{\prime } \end{array}\right]}^{T}$$

(7) $$u^{\prime }=f_{r}(u),\;u(0)=u_{0}$$

(8) $$f(u)=\left[\begin{array}{c} u_{2}\\ -\beta u_{2}-u_{1}+\cos(u_{3})+Hf_{c}\\ \eta \end{array}\right]$$

where H is the Heaviside function and $u_{0}$ is the initial condition of the rattle model.

Equation (7) is a dynamic system with discontinuities involving the discontinuous impact effects in the rattle model. Therefore, it can be rewritten as follows from Muller’s method that includes the instantaneous discontinuity of impact. Here, $\tau =\tau_{i}$ is a discontinuous moment. Let z be a state variable u of the rattle model.

(9) $$\tau_{i-1}<\tau <\tau_{i}:\;z^{\prime }=f_{i}(z),\;z(\tau_{i-1})=z(\tau_{i-1}^{+})$$

(10) $$\tau =\tau_{i}:\;0=h(z(\tau_{i}^{-}))$$

(11) $$z(\tau_{i}^{+})=g(z(\tau_{i}^{-}))$$

(12) $$\tau_{i}<\tau <\tau_{i+1}:\;z^{\prime }=f_{i+1}(z),\;z(\tau_{i})=z(\tau_{i}^{+})$$

where $\tau^{-}$ and $\tau^{+}$ denote before and after the discontinuously condition. The perturbed trajectory is given by

(13) $$\tilde{z}(\tau )=z(\tau )+\delta z(\tau )$$

(14) $${\tilde{\tau }}_{i}=\tau_{i}+\delta \tau_{i}$$

and the perturbed trajectory satisfies the following equation

(15) $${\tilde{\tau }}_{i-1}<\tau <{\tilde{\tau }}_{i}:\;{\tilde{z}}^{\prime }=f_{i}(\tilde{z}),\;\tilde{z}({\tilde{\tau }}_{i-1})=\tilde{z}({\tilde{\tau }}_{i-1}^{+})$$

(16) $$\tau ={\tilde{\tau }}_{i}:\;0=h(\tilde{z}({\tilde{\tau }}_{i}^{-}))$$

(17) $$\tilde{z}({\tilde{\tau }}_{i}^{+})=g(\tilde{z}({\tilde{\tau }}_{i}^{-}))$$

(18) $${\tilde{\tau }}_{i}<\tau <{\tilde{\tau }}_{i+1}:\;{\tilde{z}}^{\prime }=f_{i+1}(\tilde{z}),\;\tilde{z}({\tilde{\tau }}_{i})=\tilde{z}({\tilde{\tau }}_{i}^{+})$$

where each interval of discontinuities is smooth. h(z) and g(z) are the indicator function and the transition condition, respectively. The plus and minus signs denote the right- and left-sided limits, and

(19) $$\delta \tau_{i}={\tilde{\tau }}_{i}-\tau_{i}=-\frac{Dh(z_{i}^{-})\delta z_{i}^{-}}{Dh(z_{i}^{-})f_{i}(z_{i}^{-})}$$

(20) $$\delta z_{i}^{+}=Dg(z_{i}^{-})\delta z_{i}^{-}+[Dg(z_{i}^{-})f_{i}(z_{i}^{-})-f_{i+1}(z_{i}^{+})]\delta \tau_{i}$$

in which

(21) $$Dh(z_{i}^{-})={\frac{\partial h(z)}{\partial z}|}_{z=z_{i}^{-}},\;Dg(z_{i}^{-})={\frac{\partial g(z)}{\partial z}|}_{z=z_{i}^{-}}$$

are the Jacobian matrix of indicator function and transition condition at point $z_{i}^{-}$, respectively, where $z_{i}^{-}=z(\tau_{i}^{-})$ and $z_{i}^{+}=z(\tau_{i}^{+})$. For an impact oscillator with Hertz’s model of contact, the Jacobian matrix of the transition condition and indicator function becomes the following matrix [17]

(22) $$Dh(z_{i}^{-})={\left[\begin{array}{ccc} 1 & 0 & 0 \end{array}\right]}^{T},\;Dg(z_{i}^{-})=I$$

Therefore, the deviated trajectory can be written as

(23) $$\delta z^{\prime }={\frac{\partial f}{\partial z}|}_{z=z_{i}}\cdot \delta z+O_{\left(2\right)},\delta z(\tau_{0})=\delta z_{0}$$

By letting $\delta z=\left[\Phi_{\tau }(z_{0})\right]\delta z_{0}$ substitute into the perturbation Equation (23), $\delta z_{i}^{+}$ at the discontinuous region is estimated using Equation (20). The variation equation is also calculated at the same time as

(24) $$\left[{\Phi^{\prime }}_{\tau }(z_{0})\right]=[D_{z}f][\Phi_{\tau }(z_{0})],[\Phi_{\tau_{0}}(z_{0})]=[I]$$

where $\left[D_{z}f\right]$, [I], and $\left[\Phi_{\tau }(z_{0})\right]$ denote the Jacobian matrix, identity matrix, and solution of the variational equation, respectively.

For the single-mode squeak model shown in Figure 2a

(25) $$m{\ddot{x}}_{s}+c{\dot{x}}_{s}+kx_{s}=f_{0}\cos\omega_{ex}t+F_{\mu }^{x_{s}}$$

where $F_{\mu }^{x_{s}}$ is the friction force. The friction force of the single-mode squeak model is expressed as α, and h are the control parameters that determine the negative slope. $\mu_{s}$ and $\mu_{k}$ are the static and dynamic friction coefficients, respectively.

(26) $$F_{\mu }^{x_{s}}=\tanh(\alpha (V_{b}-{\dot{x}}_{s}))\left\{\mu_{k}+\left(\mu_{s}-\mu_{k}\right)\exp\left(-h\left|V_{b}-{\dot{x}}_{s}\right|\right)\right\}N$$

Using the dimensionless time $\tau =t\sqrt{k/m}$ and the coordinate transformation $x_{s}(t)=X_{s}(\tau )$, the dimensionless equation of motion can be written as

(27) $$X_{s}^{\prime \prime }+\beta X_{s}^{\prime }+X_{s}=f_{0}\cos\eta \tau +\frac{1}{k}F_{\mu }^{x_{s}}$$

where prime is the differentiation for τ(≥0), and the dimensionless parameters are defined as $\Omega =\sqrt{k/m}$, $\eta =\omega_{ex}/\Omega$, $f_{0}=F_{0}/k$, $\beta =\sqrt{c^{2}/mk}$, and $u=X_{r}/f_{0}$. Therefore, the dimensionless friction force of the single-mode squeak model can be expressed as

(28) $$f_{\mu }^{v}=\tanh\left(\alpha \left(V_{b}-v^{\prime }\right)\right)\left\{\mu_{k}+\left(\mu_{s}-\mu_{k}\right)\exp\left(-h\left|V_{b}-v^{\prime }\right|\right)\right\}n_{0}$$

where $\alpha \Omega f_{0}\to \alpha$, $V_{b}/\Omega f_{0}\to V_{b}$, $h\Omega f_{0}\to h$, and $N/kf_{0}=n_{0}$ are dimensionless parameters. Therefore, the dimensionless equation of motion for the single-mode squeak model can be written as

(29) $$v^{\prime \prime }+\beta v^{\prime }+v=\cos\eta \tau +f_{\mu }^{v}$$

The equation of motion for the single-mode squeak model is expressed in vector form

(30) $$v={\left[\begin{array}{cc} v & v^{\prime } \end{array}\right]}^{T}$$

(31) $$v^{\prime }=f_{s}(v),\;v(0)=v_{0}$$

(32) $$f_{s}(v)=\left[\begin{array}{c} v_{2}\\ -\beta v_{2}-v_{1}+\cos(v_{3})+f_{\mu }^{v}\\ \eta \end{array}\right]$$

where $v_{0}$ is the initial condition of the single-mode squeak model.

For the multi-mode squeak model shown in Figure 3

(33) $$\begin{array}{l} m{\ddot{x}}_{ms1}+\left(c+c_{1}\right){\dot{x}}_{ms1}-c_{1}{\dot{x}}_{ms2}+(k+k_{1})x_{ms1}-k_{1}x_{ms2}=F_{\mu }^{x_{ms1}}\\ m_{1}{\ddot{x}}_{ms2}-c_{1}{\dot{x}}_{ms1}+c_{1}{\dot{x}}_{ms2}-k_{1}x_{ms1}+k_{1}x_{ms2}=F_{\mu }^{x_{ms2}} \end{array}$$

where $F_{\mu }^{ms1}$ and $F_{\mu }^{ms2}$ are the friction forces acting on each mass. The friction force of the multi-mode squeak model is expressed as

(34) $$F_{\mu }^{x_{ms1}}=\tanh(\alpha (V_{b}-{\dot{x}}_{ms1}))\left\{\mu_{k}+\left(\mu_{s}-\mu_{k}\right)\exp\left(-h\left|V_{b}-{\dot{x}}_{ms1}\right|\right)\right\}N$$

(35) $$F_{\mu }^{ms2}=\tanh(\alpha (V_{b}-{\dot{x}}_{ms2}))\left\{\mu_{k}+\left(\mu_{s}-\mu_{k}\right)\exp\left(-h\left|V_{b}-{\dot{x}}_{ms2}\right|\right)\right\}N$$

Using the dimensionless time $\tau =t\sqrt{k/m}$ and the coordinate transformation $x_{ms1}(t)=X_{ms1}(\tau )$ and $x_{ms2}(t)=X_{ms2}(\tau )$, the dimensionless equation of motion can be expressed as

(36) $$\begin{array}{l} X_{ms1}^{\prime \prime }+\left(\beta +\beta_{1}\right)X_{ms1}^{\prime }-\beta_{1}X_{ms2}^{\prime }+\left(1+\gamma \right)X_{ms1}-\gamma X_{ms2}=\frac{1}{k}F_{\mu }^{x_{ms1}}\\ X_{ms2}^{\prime \prime }-\sigma \beta_{1}X_{ms1}^{\prime }+\sigma \beta_{1}X_{ms2}^{\prime }-\sigma \gamma X_{ms1}+\sigma \gamma X_{ms2}=\sigma \frac{1}{k}F_{\mu }^{x_{ms2}} \end{array}$$

where prime is the differentiation with respect to τ(≥0), and the dimensionless parameter is defined as $\Omega =\sqrt{k/m}$, $\eta =\omega_{ex}/\Omega$, $f_{0}=F_{0}/k$, $\beta =\sqrt{c^{2}/mk}$, $\beta_{1}=\sqrt{c_{1}^{2}/mk}$, $\gamma =k_{1}/k$, $\sigma =m/m_{1}$, $w=X_{ms1}/f_{0}$, and $s=X_{ms2}/f_{0}$. Therefore, the dimensionless friction force of the multi-mode squeak model can be rewritten as

(37) $$f_{\mu }^{w}=\tanh\left(\alpha \left(V_{b}-w^{\prime }\right)\right)\left\{\mu_{k}+\left(\mu_{s}-\mu_{k}\right)\exp\left(-h\left|V_{b}-w^{\prime }\right|\right)\right\}n_{0}$$

(38) $$f_{\mu }^{s}=\tanh\left(\alpha \left(V_{b}-s^{\prime }\right)\right)\left\{\mu_{k}+\left(\mu_{s}-\mu_{k}\right)\exp\left(-h\left|V_{b}-s^{\prime }\right|\right)\right\}n_{0}$$

where $\alpha \Omega f_{0}\to \alpha$, $V_{b}/\Omega f_{0}\to V_{b}$, $h\Omega f_{0}\to h$, and $N/kf_{0}=n_{0}$ are dimensionless parameters. Therefore, the dimensionless equation of motion for the simple model can be expressed as

(39) $$\begin{array}{l} w^{\prime \prime }+\left(\beta +\beta_{1}\right)w^{\prime }-\beta_{1}s^{\prime }+\left(1+\gamma \right)w-\gamma s=f_{\mu }^{w}\\ s^{\prime \prime }-\sigma \beta_{1}w^{\prime }+\sigma \beta_{1}s^{\prime }-\sigma \gamma w+\sigma \gamma s=f_{\mu }^{s} \end{array}$$

The equation of motion for the multi-mode squeak model is expressed in vector form as follows

(40) $$w={\left[\begin{array}{cccc} w & w^{\prime } & s & s^{\prime } \end{array}\right]}^{T}$$

(41) $$w^{\prime }=f_{ms}(w),\;w(0)=w_{0}$$

(42) $$f_{ms}(w)=\left[\begin{array}{c} w_{2}\\ -(\beta +\beta_{1})w_{2}+\beta_{1}s_{2}-(1+\gamma )w_{1}+\gamma s_{1}+f_{\mu }^{w}\\ s_{2}\\ \sigma \left(\beta_{1}w_{2}-\beta_{1}s_{2}+\gamma w_{1}-\gamma s_{1}+f_{\mu }^{s}\right) \end{array}\right]$$

where $w_{0}$ is the initial condition of the multi-mode squeak model.

Because the squeak models are a dynamic system without discontinuities, Equations (9)–(22) are unnecessary. Therefore, the Lyapunov exponent of the squeak model can be obtained directly from the eigenvalue of the variation Equation (24).

The Lyapunov exponents can be defined as

(43) $$\lambda_{i}=\underset{\tau \to \infty }{\lim}\frac{1}{\tau }\ln\left|m_{i}(\tau )\right|$$

where $m_{i}(t)$ are the eigenvalues of Equation (24). On the other hand, the definition cannot be used directly in the numerical calculation. If there is a considerable time, the variation equations tend to be the ill-condition [17]. Therefore, the spectrum of the Lyapunov exponent for the linearized equation was estimated using Wolf’s algorithm via the QR-factorization orthonormalization [33].

As mentioned earlier, in this study, an image of a dynamic signal was constructed based on the RP that visualized the dynamic characteristics most effectively. The recurrence is a fundamental property in a dynamic systems, and RP is a tool that visualizes the iteration of the state of the system. The corresponding RP is based on the following recurrence matrix as follows

(44) $$RP_{i,j}^{}=H\left(\varepsilon -\left\|z_{i}-z_{j}\right\|\right),i,j=1,\ldots ,N$$

where $RP_{i,j}^{}$ is called the RP or threshold RP. ‖ ⋅ ‖ is an L-2 norm; N is the measured points, and ${\left\{z_{i}\right\}}_{i=1}^{N}$ is trajectories of a system in its phase space. ε is the threshold. The threshold is a critical parameter that can be obtained differently depending on the system, but it was quantified probabilistically (ε>5σ) by Thiel et al. [34]. Therefore, an unthresholded RP without the influence of the threshold can be expressed as

(45) $$RP_{i,j}^{un}=\left\|z_{i}-z_{j}\right\|,i,j=1,\ldots ,N$$

Here the element of phase space indicates the possible state of the system for the time evolution law. In such a case, the phase space needs to be reconstructed. The method for reconstruction is generally conducted using the time delay method. Thus, the reconstructed state variable can be expressed as

(46) $$z_{i}\to {\hat{z}}_{i}=\sum_{j=1}^{m}q_{i+(j-1)\nu }e_{j}$$

where $q_{i}=q(i\Delta \tau )$, Δτ, m, ν, and $e_{j}$ are the discrete time series, sampling rate, embedding dimension, time delay, and unit vectors, respectively. The reconstruction does not change the dynamic properties, and the reconstructed phase space can be expressed through an appropriately selected embedding dimension and time delay. In general, the time delay can be selected appropriately using the mutual information method.

During time delay reconstruction, all self-crossing trajectories in the dimension $D_{A}$ of the attractor can disappear when the embedding dimension $D>2D_{A}$ is set. On the other hand, it is imperative to determine the minimum embedding dimension to minimize the Lyapunov exponents and computational calculations from a physical perspective. From Equation (46), in dimension d, $z_{r}$ is the rth nearest neighbor of z, and the square of the Euclidean distance between the two vectors is

(47) $$R_{d}^{2}(i,r)={\sum_{e=0}^{\nu -1}\left[q_{\left(i+e\nu \right)}-q_{r,(i+e\nu )}\right]}^{2}$$

Here, as the time delay embedding extends from dimension d to dimension d+1, the Euclidean distance between the rth neighbors for dimension rth can be written as follows

(48) $$R_{d+1}^{2}(i,r)=R_{d}^{2}(i,r)+{\left[q_{\left(i+e\nu \right)}-q_{r,(i+e\nu )}\right]}^{2}$$

where the error for the minimum embedding dimension can be determined from the rate of change in the Euclidean distance.

(49) $$\sqrt{\frac{R_{d+1}^{2}(i,r)-R_{d}^{2}(i,r)}{R_{d+1}^{2}(i,r)}}>R_{tol}$$

where $R_{tol}$ is the threshold. Kennel et al. [24] reported that false neighbors could be identified clearly in $R_{tol}\geq 10$. Another condition for determining false neighbors defined based on the actual value of $R_{d}(i)\equiv R_{d}(i,r=1)$ is similar to the standard variation $R_{A}$ of the attractor using finite data of the noisy signal. Thus, the Euclidean distance for the dimension d+1 becomes $R_{d+1}(i)\approx 2R_{A}$, and the second criterion for determining false neighbors can be written as

(50) $$\frac{R_{d+1}(i)}{R_{A}}>A_{tol}$$

Therefore, the minimum embedding dimension can be obtained by discriminating as false nearest neighbors (FNN) under the conditions in Equations (49) and (50).

Furthermore, the classified features were visualized through Class Activation Mapping (CAM) [35]. The procedure for CAM is as follows

(51) $$F^{k}=\sum_{x,y}^{}f_{k}(x,y)$$

where $f_{k}(x,y)$ represents the activation of the kth unit of the last convolutional layer at the spatial location (x,y). Therefore, the value obtained by Global Average Pooling (GAP) on the kth unit becomes $F^{k}$. Accordingly, the input softmax for c classes is as follows

(52) $$S_{c}=\sum_{k}^{}w_{k}^{c}\sum_{x,y}^{}f_{k}(x,y)=\sum_{k}^{}\sum_{x,y}^{}w_{k}^{c}f_{k}(x,y)$$

where $w_{k}^{c}$ is the weight corresponding to class c for kth units, the learned weight represents an optimized model for class c. The output probability of softmax for class c is as follows

(53) $$P_{c}=\frac{e^{S_{c}}}{\sum_{c}^{}e^{S_{c}}}$$

Therefore, CAM for the class is defined, and the elements on each space are given as follows

(54) $$M_{c}(x,y)=\sum_{k}^{}w_{k}^{c}f_{k}(x,y)$$

Finally, the features of the learning result using CNN can be visualized as a heat map using Equation (54).

## 3. Results

For preliminary analysis, the Rossler model, a representative chaotic system, was used [36]. The Rossler model has already been studied extensively. It is a simple chaotic vibration system because it can produce a section that always vibrates in response to a parameter change. c is selected as the control parameter. The other parameters are a=0.2 and b=0.2, and the initial condition is $q(0)={\left[\begin{array}{ccc} 1 & 1 & 1 \end{array}\right]}^{T}$.

(55) $$\dot{s}=f(\dot{s})=\left[\begin{array}{c} -s_{2}-s_{3}\\ s_{1}+as_{2}\\ b+s_{3}(s_{1}-c) \end{array}\right]$$

**Figure 4:**
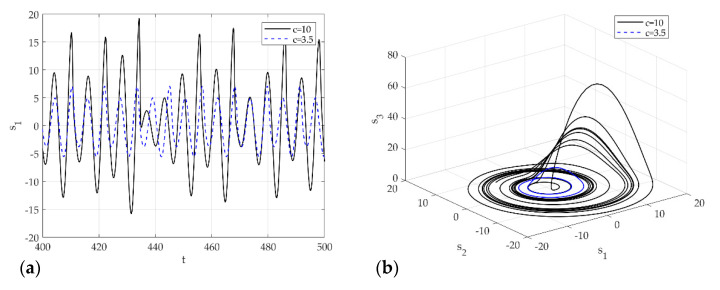
Dynamic solutions for the Rossler model for various c: (**a**) time analysis; (**b**) 3-D phase portrait.

**Figure 5:**
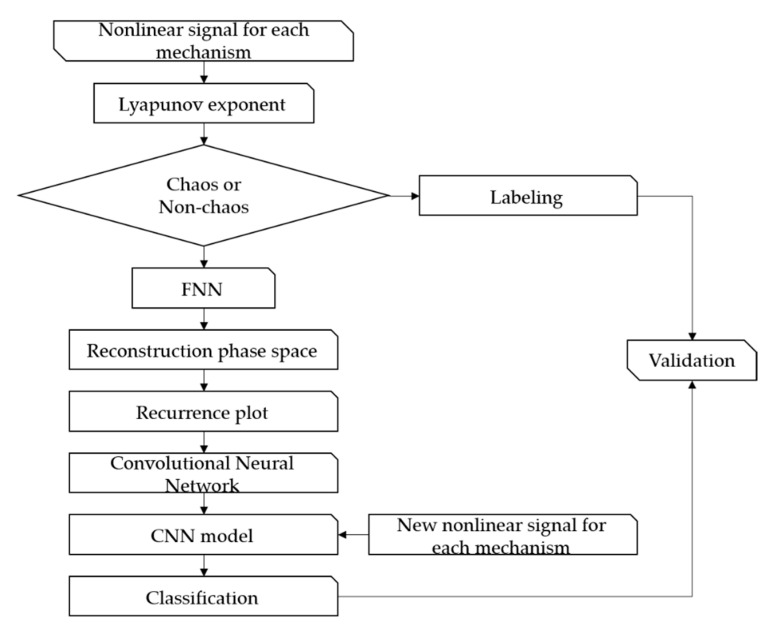
Flow diagram of the proposed methodology.

Figure 4 presents the signal $s_{1}$ for the control parameters of the Rossler model. Figure 4a is a time series analysis for c=3.5 (dash line) and c=10 (solid line). Figure 4b shows the corresponding phase space. c=3.5 shows a clear period-2 in phase space; c=10 shows the trajectory in the phase space and the aperiodic infinite trajectory in a finite boundary. On the other hand, the chaos cannot be identified clearly as a phase plot. To determine chaos, the Lyapunov exponents need to be calculated. Figure 5 presents the flow chart of the proposed methodology for applying signal classification using deep learning.

As shown in the flow diagram, the proposed method classifies the characteristics of the nonlinear vibration signals not included in learning after learning a dataset of nonlinear vibration signals composed of images using the CNN architecture. In other words, the focus of this study was to learn the vibrating signal visualized based on RP by machine learning and to distinguish between the causes of vibration, such as friction or impact and chaotic characteristics. Details of the proposed method are as follows. First, the nonlinear time series data of the parametric deterministic dynamic system was obtained by numerical analysis using the Runge–Kutta method. The Lyapunov exponent was calculated for the time series data and chaos was determined. The image visualization method of the vibration signal used the FNN algorithm to determine the embedding dimension and reconstruct the phase space. The reconstructed signal was expressed as an unthresholded RP to visualize the dynamic characteristics. Finally, the dataset composed of the visualized signals was trained by the CNN model and verified using the Lyapunov exponent.

**Figure 6:**
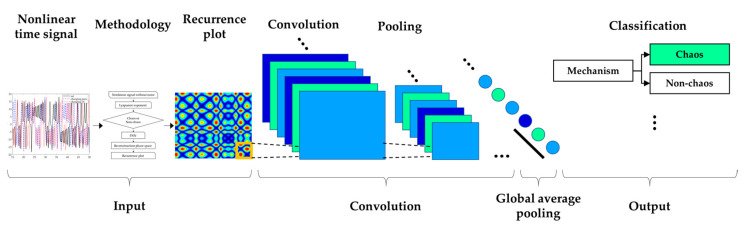
Flow diagram of CNN model.

The architecture is structured relatively simply. However, GAP was used instead of Fully Connected (FC) to activate the CAM in the last layer. GAP is relatively less accurate than FC [35]. On the other hand, the purpose of this paper was to show that even if the S&R model is chaotic, it is possible to classify it through deep learning using imaged vibration signals. Hence, the architecture was constructed simply with the aim of approximately 90% accuracy. For preliminary analysis, Table 1 lists the layer type of the CNN model, filter size, and shape of each layer. Figure 6 presents a flow diagram of the Rossler system’s CNN model.

**Figure 7:**
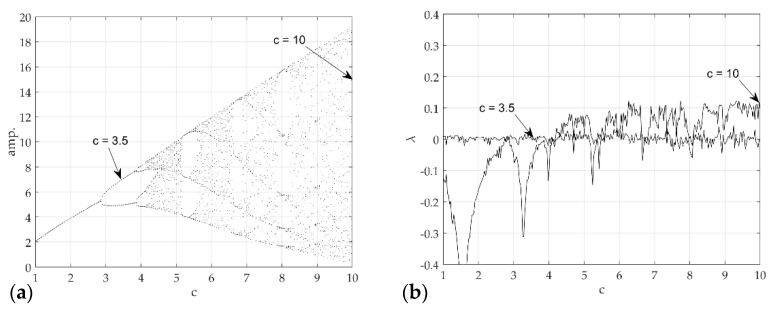
Chaotic analysis for Rossler model (**a**) bifurcation diagram of displacement (**b**) and largest Lyapunov exponent with respect to c.

**Figure 8:**
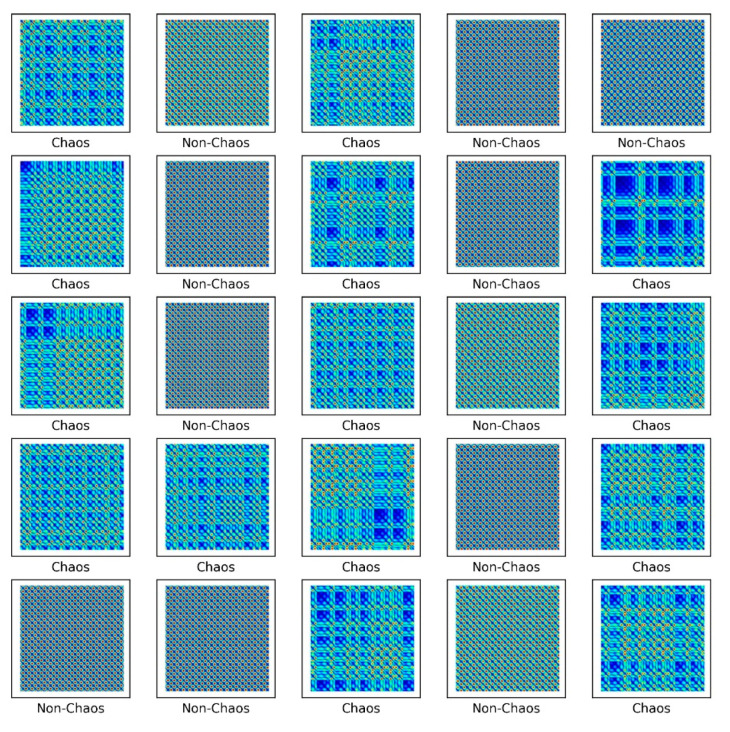
Unthresholded recurrence plot of the Rossler model.

Figure 7 presents the chaos analysis of the Rossler model. Figure 7a is a bifurcation diagram of the Rossler’s model for parameter c change, and Figure 6b shows the corresponding Lyapunov exponents. The explanation of the critical c for the Lyapunov exponents of the Rossler model has been studied extensively. Briefly, to summarize this system, the first bifurcation appears near c≈2.866, and becomes period 2. The bifurcation appears again near c≈3.86 and becomes period 4. In other words, chaos occurs as period-doubling occurs at each point. As shown in Figure 4, if the time analysis result is c=3.5, chaos is expressed as period 2, and c=10. This agrees well with the time analysis results. In 3D phase space, the Lyapunov exponent has four types of attractors: stable fixed points ($\lambda_{i}<0$, i=1,2,3), stable limit cycles ($\lambda_{1}<0$, $\lambda_{i}<0$, i=2,3), stable two-torus ($\lambda_{1}=\lambda_{2}=0$, $\lambda_{i}<0$, i=3), and strange attractors ($\lambda_{1}>0$). In the calculated system, however, only the classification of the S&R model and the existence of chaos were classified (Rossler system only distinguished between chaos and non-chaos). In other words, the strange attractor ($\lambda_{1}>0$) and dynamic characteristics of the deterministic dynamic system can be obtained from the flow of the proposed method, and an unthresholded recurrence plot was learned using CNN. Subsequently, an attempt was made to classify the signals and chaos generated by the S&R model that were not used for training. Figure 8 shows the visualized chaos and non-chaos signals for the randomly extracted Rossler model.

Image classification using CNN has developed many sophisticated models that transcend the human cognitive abilities, but the design of a sophisticated architecture was not the goal of the present study. Therefore, the architecture is composed of a simple five-level structure, as shown in Table 1. Each step includes the convolution layer, activation function, and pooling layer. The proposed model comprises five convolution layers with a 32-3 × 3 filter, 64-3 × 3 filter, 128-3 × 3 filter, 256-3 × 3 filter, and 512-3 × 3 filter in each step. As mentioned earlier, RP is a tool to visualize the recurrence characteristics of a dynamic system. The filter size was set as small as possible because the chaos system can occur within a very short interval. In addition, three max pooling layers were used. Through five convolution layers, the feature map classifies the features of the image into six classes. To use CAM, GAP was used instead of the FC layer as the last layer. Softmax was used as the activation function of the output value. One of the gradient-based optimization methods was used. The Adam optimizer is an optimization function based on the gradient descent algorithm and was used to achieve faster convergence [37]. The weight initialization is one of the fundamental problems. Incorrect weight setting causes many problems, such as convergence problems and local minima. LeCun initialization follows a Gaussian distribution and uniform distribution of weight initialization for effective backpropagation [38]. Xavier initialization sets the initial weight depending on the number of previous and next nodes [39]. This is the most generalized method, but the output value shows inefficient results when used in the ReLU function. The He initialization was developed to compensate for this [25]. For the weight initialization in the proposed CNN model, the He initialization method following a Gaussian distribution was used.

The Rossler model consisted of 3200 datasets and 200 × 200-pixel images. The intervals of the time step for the ODE and orthonormalization for the Lyapunov exponent were 0.05 and 0.1, respectively. The dataset is usually divided into three parts. The 3200 datasets were divided into 70% for the training dataset and 30% of the test dataset. The validation dataset consisted of 30% of the training dataset. Table 2 lists the dataset samples used for training. The errors due to sequential datasets were removed by shuffling the dataset because the images are generated sequentially for parameter analysis. Deep learning requires a high-performance computer. The hardware used was a GPU machine (NFEC-2021-01-267120, Future Automotive Intelligent Electronics Core Technology Center) with NVIDIA GPU V100.

**Figure 9:**
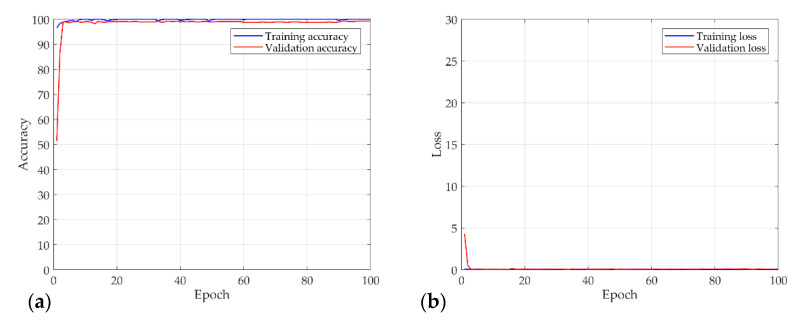
Results of the numerical experiment for the Rossler model: (**a**) accuracy; (**b**) and loss curves over 100 epochs.

Figure 9 shows the results of a numerical experiment for the proposed procedure. Chaos characteristics were found in the training dataset for 2240. At the same time, it was verified through 560 validation data in each epoch. After that, the tests were performed on 1200 testing datasets on the trained CNN model. The batch size was set to 10, and the learning rate of the optimization function was 0.0001. Figure 9a,b shows the accuracy and loss function of the training data and validation data for each epoch. As shown in the learning result, the accuracy showed a logarithmic function and converged to approximately 100, and the loss also showed a negative exponential function and converged close to zero. The accuracy and loss of validation data and the training data almost coincide, suggesting that the training proceeded well without overfitting. This suggests that the proposed procedure detects the chaos characteristics of the Rossler model well. In addition, 1200 testing datasets that were not used for training were also classified with 99% accuracy.

**Figure 10:**
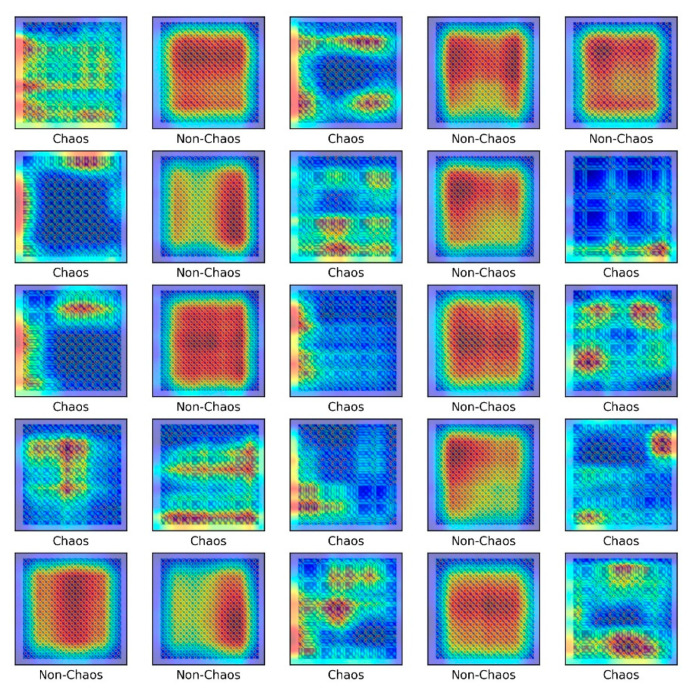
Class activation mapping for the Rossler model.

Figure 10 shows the heat map using CAM. The heat map shows the spatial importance for each class, and red is the most important part. As shown in the heat map, when the iteration of the Rossler model is non-chaos, the characteristics of the image tend to be uniform and symmetrical, and the chaos is irregular.

In the Rossler model, the cycle is long and clearly expressed for the set control parameters. Therefore, the visualized repetition was distinguished easily. On the other hand, the S&R model, which includes the friction force and impact force, contains extreme nonlinearities so that the repeatability can be very complex. Here, because the rattle problem contains discontinuities, the Lyapunov exponent was calculated by considering Muller’s method. The squeak problem has a continuity, including creep, so the Lyapunov was calculated as a continuous dynamic problem.

**Figure 11:**
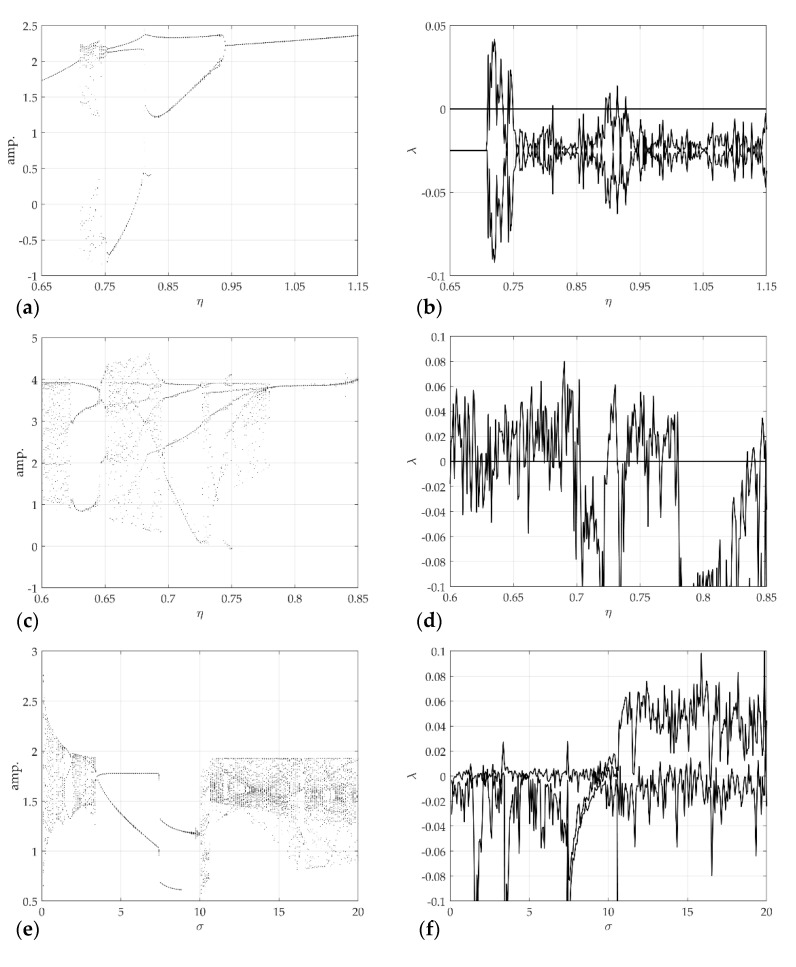
Chaotic analysis for the S&R model: (**a**) bifurcation diagram of displacements; (**b**) largest Lyapunov exponent, for rattle model with respect to η; (**c**) bifurcation diagram of displacements; (**d**) largest Lyapunov exponent, for single-mode squeak model with respect to η; (**e**) bifurcation diagram of displacements; (**f**) largest Lyapunov exponent, for multi-modes squeak model with respect to σ.

Figure 11 shows the results of chaotic analysis for the model control parameters corresponding to each model. Figure 11a is the calculation result of the largest Lyapunov exponent of the rattle model, and Figure 11b shows the corresponding bifurcation diagram. The other parameters and initial conditions are r=2, ξ=0.05, $k_{h}=100$, and $u_{0}={\left[\begin{array}{ccc} 0.4 & -1.1 & 0 \end{array}\right]}^{T}$. Figure 11c,d shows the Lyapunov exponent calculation result for the control parameter of the single-mode squeak model and the corresponding bifurcation diagram. The other parameters and initial conditions are V=2, $n_{0}=2.5$, β=0.002, $\mu_{s}=0.5$, $\mu_{k}=0.1$, h=1, α=65, and $v_{0}={\left[\begin{array}{ccc} 0.1 & 1.2 & 0 \end{array}\right]}^{T}$. Figure 11e,f presents the Lyapunov exponent calculation result and bifurcation diagram for the control parameter of the multi-modes squeak model. Other parameters and initial conditions were V=1, $n_{0}=2.5$, $\beta =\beta_{1}=0$, $\mu_{s}=0.5$, $\mu_{k}=0.3$, h=1, α=65, γ=0.1, and $w_{0}={\left[\begin{array}{cccc} 0.1 & 0.1 & 0.1 & 1.1 \end{array}\right]}^{T}$. In this study, only each model and chaotic characteristics were distinguished, so other detailed types of attractors were not considered. The analysis results show that the S&R model changes with extreme nonlinearity in the largest Lyapunov exponent for the change in the control parameter.

**Figure 12:**
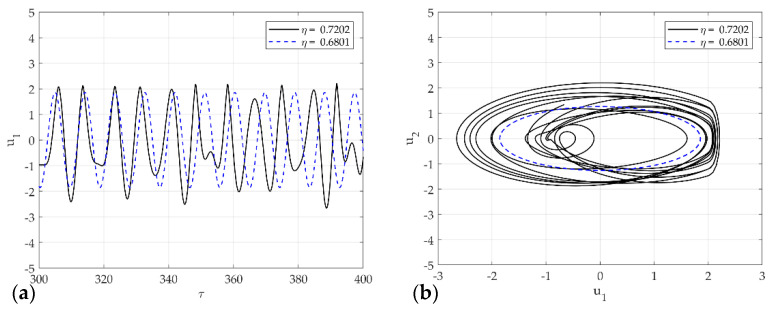
Dynamic solutions for the rattle model for various η: (**a**) time analysis; (**b**) phase portrait corresponding to (**a**).

**Figure 13:**
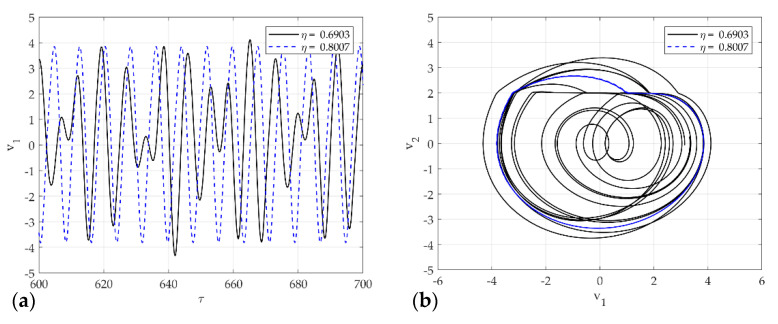
Dynamic solutions for single-mode squeak model for various η: (**a**) time analysis; (**b**) phase portrait corresponding to (**a**).

**Figure 14:**
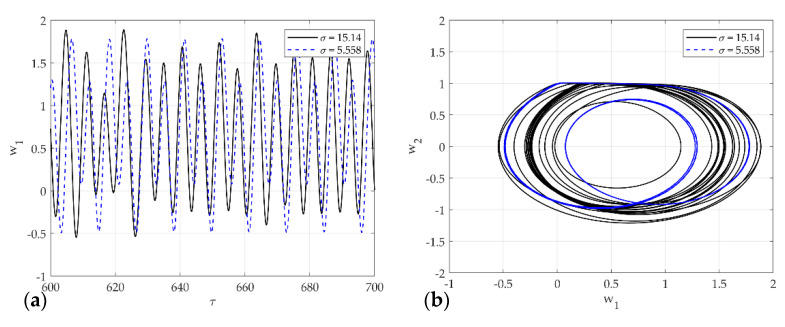
Dynamic solutions for single-mode squeak model for various σ: (**a**) time analysis; (**b**) phase portrait corresponding to (**a**).

As mentioned earlier, Figure 12, Figure 13 and Figure 14 presents the representative attractors of each system divided into chaos and non-chaos, and show the rattle, single-mode squeak, and multi-mode squeak models, respectively. Figure 12a,b shows the time series plot and phase portrait of displacement for the rattle model in η=0.7202 and η=0.6801. The dotted line oscillates constantly, and the solid line vibrates with an irregular amplitude. In phase space at η=0.6801, it produces one stable limit cycle without impact and oscillates stably. On the other hand, in η=0.7202, the system includes impact, and the trajectory appears without a specific period. In other words, it expresses chaos.

Figure 13a,b shows the time series plot and phase portrait of displacement for the single-mode squeak model in η=0.8007 and η=0.6903. The dotted line oscillates constantly, and the solid line vibrates with an irregular amplitude. In the phase plot, the flat phase means the stick phase in the stick-slip. When η=0.8007 produces an unstable limit cycle in phase space, it vibrates unstably for 1 period within the limit cycle. On the other hand, in η=0.6903, the system produces an unstable limit cycle and generates chaos without a constant cycle.

Figure 14a,b shows the time series plot and phase portrait of displacement for the multi-mode squeak model in σ=15.14 and σ=5.558. The dotted line constantly oscillates with two amplitudes, and the solid line oscillates with an irregular amplitude. In phase space at σ=15.14, it vibrates unstably for two periods within the limit cycle. On the other hand, at σ=5.558, the system generates an unstable limit cycle and generates chaos.

**Figure 15:**
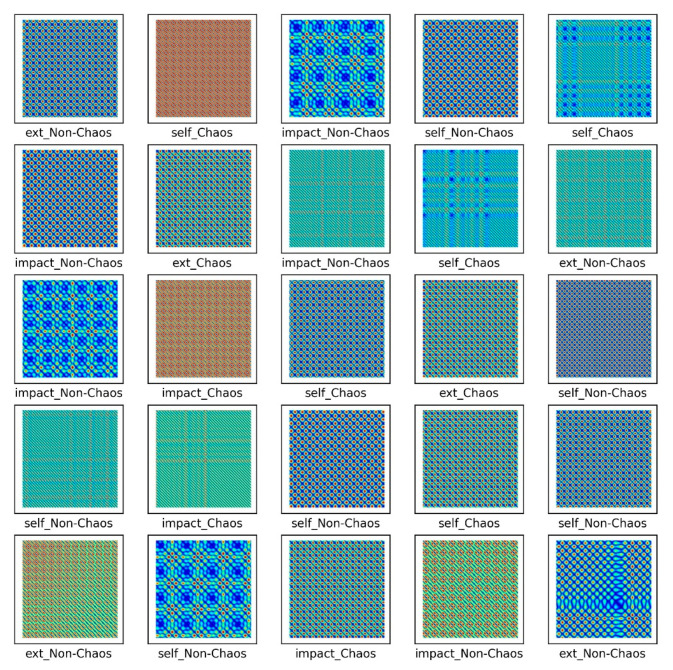
Unthresholded RP for the S&R model.

The S&R model was classified into six classes to distinguish between chaos and non-chaos, and an unthresholded RP was shown. Here, the six classes were divided into the rattle, single-mode squeak and multi-modes squeak, and chaos and non-chaos for each model. Table 3 lists the dataset sample, and Figure 15 presents a part of the training dataset.

As shown in Figure 15, images by chaos and images by non-chaos are always vibrating, so repetition appears in a complex form. Six thousand data were used in the total dataset and consisted of 200 × 200-pixel images. To escape the local minima and converge to a lower loss, and prevent overfitting, the learning rate was adjusted for each specific step using a callback function. Each step consisted of five convolution layers with 32-2 × 2 filter, 64-2 × 2 filter, 128-2 × 2 filter, 256-2 × 2 filter, and 512-2 × 2 filter.

**Figure 16:**
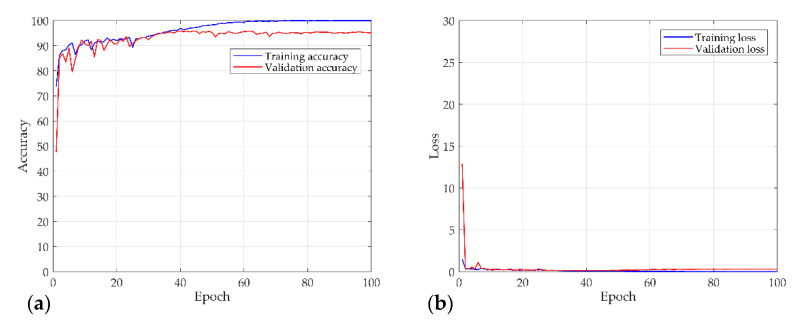
Results of the numerical experiment for the S&R model: (**a**) accuracy; (**b**) and loss curves over 100 epochs.

**Figure 17:**
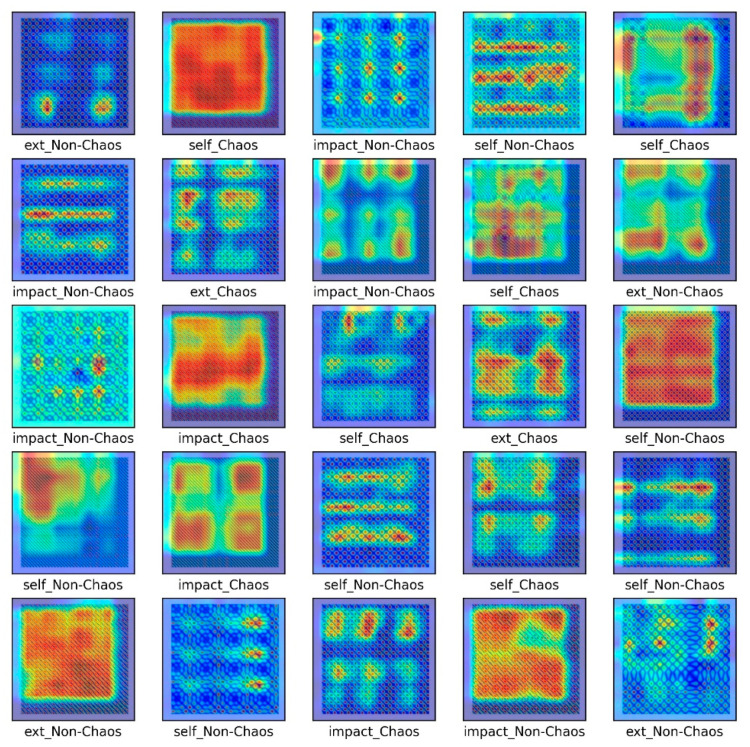
Class activation map for the S&R model.

Figure 16 shows the results of the numerical experiment. The validation loss and accuracy do not decrease until 20 epochs but escape from the local minima by adjusting the learning rate. This shows that the learning rate is adjusted again at 70 epochs and converges with a certain accuracy and loss. In the final test, the model of each system and the chaos problem were classified with approximately 90% accuracy. Figure 17 shows the characteristics of the nonlinear vibration signal of each system for data extracted randomly by CAM. The most important characteristic of the recurrence plot is on the main diagonal line, meaning $RP_{i,j}^{un}=RP_{j,i}^{un}$. If $z_{i}\neq z_{j}$, an aperiodic pattern representing chaos may appear. As shown in Figure 17, macroscopically, the pattern is almost similar, but if the measurement length is long or the period is fast, a very small aperiodic pattern can be indicated. In other words, the CAM results in the first row and second column appear macroscopically similar, but physically show completely different characteristics.

The areas of difference for the recurrence characteristics for each model were detected successfully using deep learning. The proposed procedure can classify the nonlinear vibration characteristics generated in the mechanical system with high accuracy. In other words, the causes of complex signals can be classified due to nonlinear vibrations generated in mechanical systems with high accuracy. This shows that the characteristics of the complex signals, such as BSR noise, that humans cannot recognize can be classified with high accuracy by a CNN.

## 4. Discussion and Future Work

Visualization was performed with the proposed method for the vibration signals with extreme nonlinearity occurring in different models. The CNN was used to classify the S&R model and its chaotic characteristics. This result was verified by calculating the Lyapunov exponent for each model. The chaotic characteristics can distinguish the signals generated in a deterministic system by calculating the Lyapunov exponent, but calculating this is very complex. The signal visualization method analyzes the dynamic signals, but signals containing nonlinearity are complicated for engineers to analyze. In other words, complex signals are difficult to classify by human cognitive ability. Therefore, a procedure for classifying S&R models, including chaos and non-chaos, was proposed and verified with approximately 91% accuracy using a simple CNN model. In future work, we will study more complex models to analyze signals with added noise. It will also conduct detailed experimental studies.

## Figures and Tables

**Table 1:** CNN model summary.

Layer (Type)	Output Shape	Param #
Conv2d	(None, 200, 200, 32)	896
Batch normalization	(None, 200, 200, 32)	128
Max pooling 2d	(None, 100, 100, 32)	0
Conv2d_1	(None, 100, 100, 64)	18,496
Batch normalization_1	(None, 100, 100, 64)	256
Max pooling 2d_1	(None, 50, 50, 64)	0
Conv2d_2	(None, 50, 50, 128)	73,856
Batch normalization_2	(None, 50, 50, 128)	512
Max pooling 2d_2	(None, 25, 25, 128)	0
Conv2d_3	(None, 25, 25, 256)	295,168
Batch normalization_3	(None, 25, 25, 256)	1024
Max pooling 2d_3	(None, 12, 12, 256)	0
Conv2d_4	(None, 12, 12, 512)	1,180,160
Global Average Pooling 2d	(None, 512)	0
Dense	(None, 2)	1026

**Table 2:** Dataset split ratio for the Rossler model.

Data	Percentage	Number of Samples
Training	56%	2240
Validation	14%	560
Testing	30%	1200

**Table 3:** Dataset split ratio for the S&R model.

Data	Percentage	Number of Samples
Training	56%	4160
Validation	14%	1040
Testing	30%	1800
